# Factors influencing patient decision-making on a multimodal precision medicine algorithm for depression: a qualitative European multicentre study of the PROMPT consortium

**DOI:** 10.3389/fpsyt.2025.1713160

**Published:** 2025-12-16

**Authors:** Rosa Glaser, Johannes C. S. Zang, Britta Kelch, Silke Jörgens, Inga Stonner, Viktor T. H. Wahner, Ewa Ferensztajn-Rochowiak, Dobrochna Kopeć, Martina Contu, Pasquale Paribello, Marco Pinna, Mara Dierssen, Massimo Gennarelli, Mirko Manchia, Alessandra Minelli, Júlia Perera Bel, Marie-Claude Potier, Filip Rybakowski, Ferran Sanz, Alessio Squassina, on behalf of PROMPT consortium, Bernhard T. Baune

**Affiliations:** 1 Department of Psychiatry, University of Münster, Münster, Germany; 2 Department of General Internal Medicine and Psychosomatic, University Hospital Heidelberg, Heidelberg, Germany; 3 Department Hamm 2, Hochschule Hamm-Lippstadt, Hamm, Germany; 4 Department of Adult Psychiatry, Poznan University of Medical Sciences, Poznan, Poland; 5 Section of Psychiatry, Department of Medical Sciences and Public Health, University of Cagliari, Cagliari, Italy; 6 Centre for Genomic Regulation (CRG), The Barcelona Institute of Science and Technology, Barcelona, Spain; 7 Department of Molecular and Translational Medicine, University of Brescia, Brescia, Italy; 8 Genetics Unit, San Giovanni di Dio Fatebenefratelli Center (IRCCS), Brescia, Italy; 9 Department of Pharmacology, Dalhousie University, Halifax, NS, Canada; 10 Unit of Clinical Psychiatry, Department of Medicine, University Hospital Agency of Cagliari, Cagliari, Italy; 11 Research Programme on Biomedical Informatics (GRIB), Hospital del Mar Research Institute (IMIM), Barcelona, Spain; 12 Paris Brain Institute (ICM), National Centre for Scientific Research (CNRS), Paris, France; 13 Department of Medicine and Life Sciences, Universitat Pompeu Fabra, Barcelona, Spain; 14 Section of Neuroscience and Clinical Pharmacology, Department of Biomedical Sciences, University of Cagliari, Cagliari, Italy; 15 Department of Psychiatry, Dalhousie University, Halifax, NS, Canada; 16 Florey Institute of Neuroscience and Mental Health, Parkville, Vic, Australia; 17 Department of Psychiatry, University of Melbourne, Parkville, Vic, Australia

**Keywords:** precision medicine, precision psychiatry, predictive algorithm, treatment resistance, antidepressants, focus groups, major depressive disorder

## Abstract

**Background:**

Precision medicine promises to improve treatment outcomes by tailoring interventions to patients’ individual characteristics. However, the use of precision medicine tools requires patient acceptance, which remains underexplored. This qualitative study investigated factors influencing patient perspectives on a multimodal precision medicine algorithm to predict antidepressant response in patients suffering from Major Depressive Disorder (MDD).

**Methods:**

To explore patients’ perspectives on the use of a multimodal algorithm, semi-structured focus groups were conducted with 44 patients diagnosed with moderate to severe depression across three European sites (Germany, Poland, Italy) in the PROMPT study. Discussions were transcribed and translated into English for analysis. A qualitative, structured content analysis approach was then used to analyse the data.

**Results:**

Patients’ perspectives on using a multimodal algorithm in MDD revealed a complex interplay of decision-making factors: while perceived clinical benefits, such as a reduction in trial-and-error prescribing and reassurance, promoted acceptance of the algorithm, concerns about cost, waiting time and the emotional impact of unfavourable results tended to discourage acceptance. Patients’ general beliefs about mental illness and its treatment shaped their attitudes toward the application of the algorithm. Many participants emphasised the importance of trust in physicians and preferred testing within the context of an established therapeutic relationship. Misconceptions about the algorithm’s accuracy and capabilities, and fears of medical reductionism, were common.

**Conclusions:**

While patients are open to the use of a multimodal precision medicine algorithm for MDD, they emphasised the need for individualised, transparent communication and emotional support. The results highlight the importance of patient-centred communication strategies and guidelines for the ethical implementation of precision psychiatry.

## 1 Introduction

MDD remains a leading cause of disability, affecting approximately 300 million individuals worldwide (1). Despite available antidepressant treatment, up to 30% of patients develop treatment-resistant depression (TRD). Commonly defined as an inadequate response to at least two antidepressant trials (2, 3), TRD is associated with severe functional impairment (4, 5), increased suicidality (6, 7) and increased healthcare costs (7, 8). The current trial-and-error approach to antidepressant selection prolongs suffering and decreases treatment success (2, 9), driving interest in more personalised, effective care.

Precision medicine, and precision psychiatry in particular, offers a promising way of optimising antidepressant therapy. By incorporating clinical, genetic, environmental, and psychosocial factors, this approach aims to predict antidepressant response, enabling earlier and more targeted treatment, particularly at the initiation of pharmacotherapy, to reduce the risk of treatment resistance (4).

This study was conducted as part of the European research project, PROMPT (Towards Precision Medicine for the Prediction of Treatment Response in Major Depressive Disorder through the Stratification of Combined Clinical and Omics Signatures), which is funded by ERA PerMed. The aim of PROMPT is to develop and evaluate a novel, multimodal precision medicine algorithm that combines genetic, clinical and sex-related data in order to estimate an individual’s likelihood of responding to antidepressant treatment for MDD (10). Unlike traditional pharmacogenetic tests, which focus on drug metabolism, this algorithm generates probabilistic predictions based on a broader range of input variables. The long-term goal is to support early clinical decision-making and prevent prolonged ineffective treatment.

To ensure responsible use, it is essential to understand patients’ perspectives on this new generation of clinical tools (10). Although precision medicine approaches are becoming more common, successfully integrating them into psychiatric care depends not only on clinical utility but also on patient acceptance (11). To date, research on patients’ perspectives of precision psychiatry has focused primarily on pharmacogenetics, with broader approaches receiving little attention. Although previous studies on genetic and pharmacogenetic testing in psychiatry have highlighted important aspects, such as ethical, financial, and psychological concerns (12–15), they may not have addressed the unique interpretative and communicative challenges of a more complex tool like the PROMPT multimodal algorithm. Moreover, much of the existing research has focused on the perspectives of healthcare professionals or has been conducted outside of Europe (16). Finally, while previous studies based on surveys of genetic and pharmacogenetic testing often provided valuable insights, they may fall short of capturing the complexity of individual decision-making due to their hypothesis-driven nature (17). Qualitative approaches, such as focus group discussions for example, can provide deeper insights into patients’ motivations, expectations, and reservations (18).

To address these gaps, this study explores factors shaping patient decision-making regarding the PROMPT multimodal precision medicine algorithm. Based on focus group data from Germany, Italy, and Poland, we explored the perspectives, concerns, and expectations of patients regarding the use of a multimodal algorithm in psychiatric care. Although this study does not examine a pharmacogenetic test specifically, we refer to existing pharmacogenetic research throughout the manuscript as a reference point since up to date these represent the most thoroughly studied precision medicine applications in psychiatry. The findings offer practical guidance for the ethical and effective implementation of the PROMPT multimodal algorithm, and inform broader efforts to integrate precision medicine into psychiatric care.

## 2 Methods

### 2.1 Study design

We conducted a cross-sectional qualitative study using focus group interviews to explore patients’ perspectives. This method is particularly suited to uncovering complex and sensitive issues through shared experiences, thereby offering genuine insights into decision-making, associated motives, concerns, and hopes (19).

### 2.2 Participant recruitment

Patients currently in treatment for MDD at one of the three PROMT study sites – Münster (Germany), Poznań (Poland), and Cagliari (Italy) were invited to participate in the focus groups. All participants had a diagnosis of moderate to severe depression. To ensure effective moderation and participation, we aimed to include 10–15 patients per site, forming groups of 4–5 individuals. In total, 11 focus groups were conducted, involving 44 patients. Focus groups conducted in Cagliari included only outpatients, Münster groups only inpatients, and Poznań groups a mix of both.

### 2.3 Data collection

Focus groups were conducted at each site in the respective national language from April to September 2023. Before the discussions, participants completed brief questionnaires covering socio-demographic and clinical information. Two researchers, with backgrounds in medicine, psychology, or health sciences, facilitated each session – one moderating the discussion and the other taking field notes on participant dynamics while prompting further discussion as needed. The discussions were conducted as semi-structured interviews following a pre-developed interview guide. This guide is based on existing literature on pharmacogenetic testing, patient expectations, and ethical concerns related to precision psychiatry (16, 20–22). The discussions were divided into two parts: First, the patients were asked to report on their personal experiences with MDD and its treatment and answer questions about the perceived causes of their depression, the treatment history, and the role of antidepressants in particular. All patients were then introduced to the multimodal precision medicine algorithm developed as part of the PROMPT study using a chart to depict the algorithm application procedure and its outcome and subsequently asked to consider its potential personal relevance. This section of the focus group guide included questions about their openness to using the algorithm, their decision-making criteria, their emotional reactions to hypothetical results, and their more general concerns, such as privacy, cost, and moral beliefs (see **Supplementary Table 1** for an overview of the interview guide). All sessions, lasting up to two hours, were audio-recorded and transcribed verbatim. Transcripts were translated into English using ‘DeepL translator’ and subsequently verified for accuracy by native-speaking members of the research team.

### 2.4 Data analysis

We applied qualitative description using qualitative content structuring content analysis with the analysis software ‘MAXQDA’ (23). Initially, broad categories were established deductively from the interview guide. Two researchers independently reviewed the first focus group transcript from each site to inductively identify additional codes, leading to the development of a preliminary coding framework. One researcher then coded the remaining transcripts, refining the framework as needed after consultation. To ensure reliability, three team members not involved in the coding process descriptively summarised the coded segments, prompting further refinements. Through the development of code memos, the segments of each category were then examined in the context of the research questions and were systematically integrated.

Although inter-coder agreement was not formally calculated, any discrepancies in coding were discussed until a consensus was reached. This reflexive process, supported by iterative framework refinement and team consultation, ensured analytical rigour.

## 3 Results

A total of 44 individuals participated in the focus groups across Germany (n = 16), Italy (n = 13), and Poland (n = 15). The overall mean age was 46.7 years (SD = 12.7), with a range from 21 to 73 years. The average age of first depressive episode was 29.1 years (SD = 13.5). The majority of participants identified as female (63.6%). At the time of data collection, most participants (79.5%) reported experiencing a current depressive episode. On a seven-point Likert scale ranging from 1 (not at all burdened) to 7 (very strongly burdened), the median reported burden was 5.5 (interquartile range (IQR) = 4–7) and the mean was 5.1 (standard deviation (SD) = 1.7), indicating a generally high subjective burden across groups. Full sociodemographic details are presented in **Table 1**.

**Table 1:** Demographic characteristics of focus group participants in the PROMPT study.

Variable	Germany (N = 16)	Italy (N = 13)	Poland (N = 15)	Overall (N = 44)
Age, mean (SD)	43.8 (15.1) (Min = 21; Max = 73)	52.1 (13) (Min = 26; Max = 68)	45.2 (10.9) (Min = 28; Max = 67)	46.7 (12.7) (Min = 21; Max = 73)
Gender, n (%)				
*Female*	7 (15.9)	10 (22.7)	11 (25)	28 (63.6)
*Male*	9 (20.5)	3 (6.8)	4 (9.1)	16 (36.4)
Religious, n (%)				
*Yes*	5 (11.4)	9 (20.5)	6 (13.6)	20 (45.5)
*No*	10 (22.7)	3 (6.8)	8 (18.2)	21 (47.7)
*Not stated*	1 (2.3)	1 (2.3)	1 (2.3)	3 (6.8)
Age at first onset, mean (SD)	30.8 (15.1)	24.6 (13.19)	31.1 (12.1)	29.1 (13.5)
Current depressive episode, n (%)				
*Yes*	13 (29.5)	10 (22.7)	12 (27.3)	35 (79.5)
*No*	3 (6.8)	3 (6.8)	2 (4.5)	8 (18.2)
*Not stated*	–	–	1 (2.3)	1 (2.3)
Experienced burden				
*mean (SD)*	5.5 (1.41)	4.6 (2)	5.13 (1.7)	5.1 (1.7)
*Mdn (IQR)*	5.5 (4 - 7)	6 (3 - 6)	5 (4 - 6.5)	5.5 (4 - 7)
Occupational status, n (%)				
*Permanent position*	6 (13.6)	6 (13.6)	8 (18.2)	20 (45.5)
*Unemployed*	5 (11.4)	2 (4.5)	2 (4.5)	9 (20.5)
*Self employed*	–	2 (4.5)	1 (2.3)	3 (6.8)
*Retired*	3 (6.8)	–	1 (2.3)	4 (9.1)
*Houseman/Housewife*	–	–	2 (4.5)	2 (4.5)
*Student*	1 (2.3)	–	–	1 (2.3)
*Something else*	1 (2.3)	–	–	1 (2.3)
*Not stated*	–	3 (6.8)	1 (2.3)	4 (9.1)

M, mean; SD, standard deviation; Mdn, median; IQR, interquartile range (25th–75th percentile). *7-point Likert scale Item (1, not at all burdened; 7, very strongly burdened)

**Figure 1:**
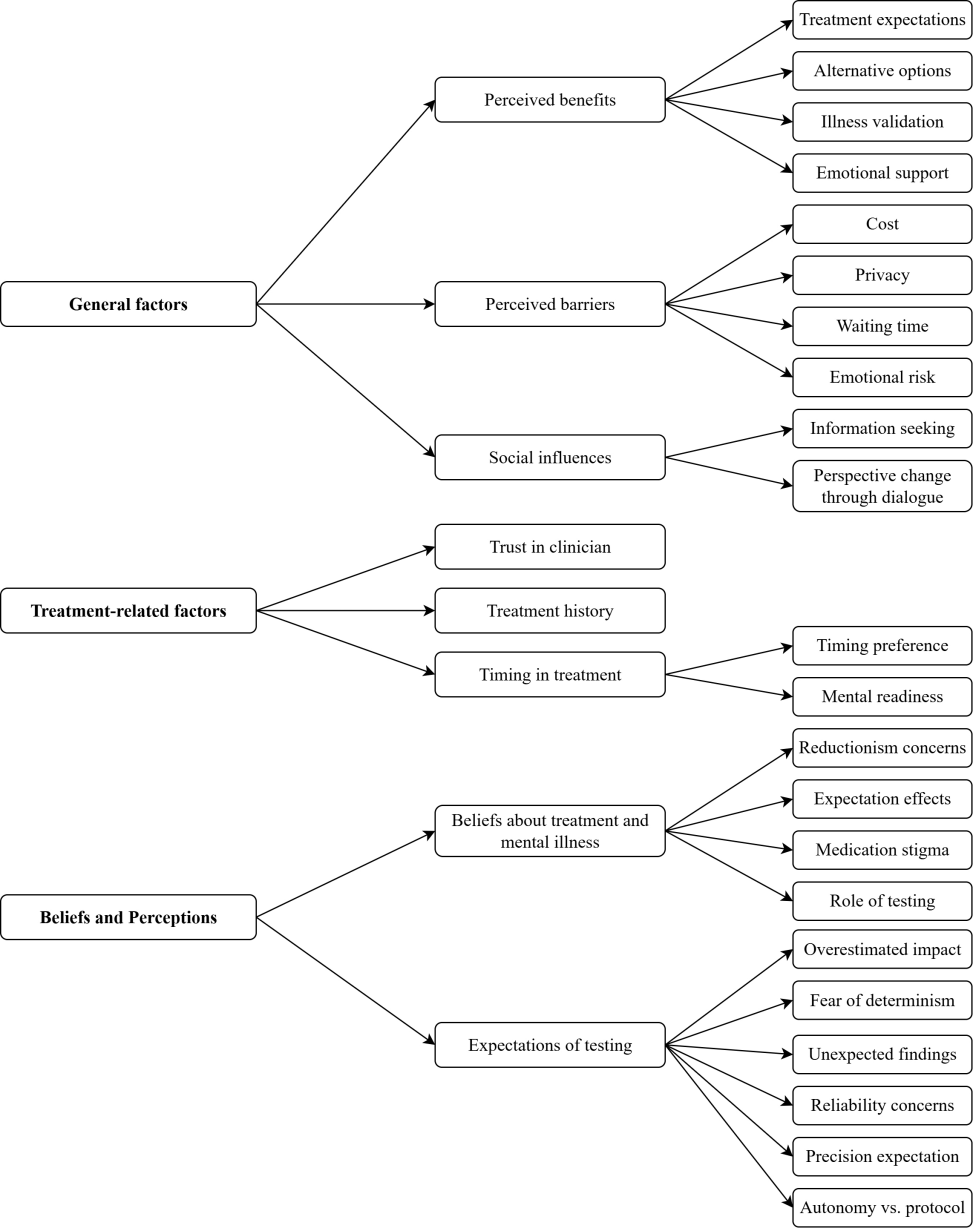
Patient-reported factors influencing decisions on algorithm use, grouped by three thematic domains.

Overall, factors from three main areas were identified: general decision-making factors; treatment-related decision factors; and underlying perceptions and beliefs about mental illness and testing. **Figure 1** provides a visual summary of all identified factors and illustrates how they are grouped thematically. In the following, we will present the identified factors in detail. An overview of the results and the recommendations derived from them can be found in **Table 2**.

**Table 2:** Patient-reported decision-making factors and derived clinical recommendations for the implementation of the PROMPT multimodal algorithm in depression treatment.

Factor	Theme	Patient perspective	Recommendation
General factors			
	***Perceived benefits***		
	Perceived clinical utility	Hope for faster, more effective, and less burdensome treatment	Clearly explain that the algorithm estimates probabilities, not guarantees; set realistic expectations about clinical benefits
	Alternative treatment options	Desire for comprehensive care, not limited to medication	Integrate results into a broader plan including psychotherapy, psychosocial support, and lifestyle modifications
	Illness validation	Feeling seen and legitimised by a biological explanation	Acknowledge the validating effect while emphasising that all forms of distress are real and deserving of care
	Emotional reassurance	Algorithm seen as offering guidance and reassurance	Use the results to support emotional coping, but emphasise the inherent uncertainty of probabilistic tools
	***Perceived barriers***		
	Financial concerns	High costs and coverage gaps discourage uptake	Advocate for insurance coverage and clearly communicate potential financial implications
	Privacy concerns	Concerns about misuse of genetic data, especially internationally	Provide transparent, country-specific information about data storage, access, and protections
	Waiting time	Concern over delays in starting treatment	Set clear expectations about timelines; offer interim support during waiting periods
	Emotional risk	Relief from positive results; anxiety or despair from negative ones	Prepare patients for the emotional impact of algorithm-based predictions; offer emotional support before and after delivering results
	***Social influences***		
	Information seeking	Importance of transparent communication	Offer early, reliable, and accessible information; provide space for questions
	Perspective change through dialogue	Opinions shifted with time and discussion	Facilitate opportunities for reflection and open exchange (e.g. peer groups or shared decision-making); allow time and flexibility to revisit decisions
Treatment-related factors			
	***Trust in clinician***	Trust in clinician shaped willingness to test	Offer the algorithm within established therapeutic relationships; use shared decision-making
	***Treatment history***	Openness higher after treatment failures and lower with treatment success	Use treatment history to guide timing; offer the algorithm as a supportive next step after treatment setbacks
	***Timing in treatment***		
	Timing preference	Mixed views on when to offer testing; early testing seed as efficient by some, but demotivation by others	Offer testing flexibly, respecting individual timing preferences
	Mental readiness	Willingness to engage varied with mental state; some felt unable to decide during acute phases	Consider mental capacity and involve support persons when needed
Beliefs and perceptions			
	***Underlying beliefs about treatment and mental illness***		
	Reductionism concerns	Fear that results may overshadow lived experience	Frame testing as one part of a broader, integrative treatment approach
	Expectation effects	Beliefs about placebo/nocebo effects influencing outcome	Frame results in a non-deterministic way; highlight the role of mindset in treatment success
	Medication stigma	Expectation that results could validate the illness and counter stigma	Acknowledge this perceived value while avoiding overemphasis on biological models of mental illness
	Integrated use of testing	Worry that testing could replace clinical judgment or be used in isolation	Emphasise that testing is a tool that supports, rather than replaces, clinical assessment
	***Perceptions and expectations of testing***		
	Overestimated predictive accuracy	Perception of the algorithm as diagnostic or overly precise	Clearly communicate the algorithm’s purpose, limitations, and probabilistic output
	Fear determinism	Concerns that results could lead to exclusion of treatment options or neglect of individual variation	Emphasise that test results are not deterministic and do not preclude other treatments
	Unexpected findings	Expectation that the algorithm could detect unrelated illnesses	Clearly communicate that the algorithm predicts pharmacological response only
	Reliability concerns	High accuracy expected, with individual variation in acceptable thresholds	Communicate the reliability of the algorithm transparently and explore patient-specific comfort levels with uncertainty
	Ambivalence toward scientific precision	Discomfort with mechanical models of illness	Frame biological information within a holistic view of mental health and personal identity
	Autonomy vs. standardisation	Standardisation viewed as threat or relief, depending on perspective	Present the algorithm as an optional tool and support shared decision-making

### 3.1 General decision-making factors

Patients generally responded with immediate hope and optimism to the prospect of a multimodal precision medicine algorithm able to predict individual response to antidepressant medication. Those who had undergone prolonged and unsuccessful medication trials believed that the multimodal algorithm could reduce the inefficiencies of the current trial-and-error approach to prescribing antidepressants. They described trial-and-error treatment as stressful and burdensome, highlighting the physical and emotional toll of medication changes, side effects, and uncertainty, which added significant distress during depressive episodes. Many patients regretted not having access to such a predictive tool earlier, as they believed it would have shortened their suffering and minimised the disruption to their personal and professional lives:

“I would have been better off before, avoiding neglecting everything – work, home, friendships, family. For too long I had disinterest in everything and everyone, how bad.” [CAG-FG2-P2]

For some, a negative result predicting nonresponse to antidepressants was not seen as a failure but rather as an opportunity to explore non-drug alternatives. These included psychotherapy, lifestyle changes or even complementary approaches such as Ayurveda or Traditional Chinese Medicine. However, many patients lacked clarity about the alternatives available, which reduced their motivation to participate. Some patients appreciated the ability of the multimodal algorithm to provide objective biological evidence to support treatment decisions, as self-assessments are always fluctuating:

“It is certainly more precise [as] I judge myself [ … ] that today I could move mountains, and tomorrow let them stand quietly.” [POZ-FG1-P1]

Even more, some saw genetic markers as a way to counter scepticism about mental illness, making it easier for both patients and society to accept depression as a legitimate medical condition. The inclusion of biological data would enhance the credibility of psychiatric diagnoses:

“There is such a group of people who deny mental illness. And here there will be such evidence, (…) I would say, of blood results.” [POZ-FG5-P4]

Many participants expressed that a positive result predicting response to antidepressants would bring hope, reassurance, and relief – especially after previous frustrations with trial-and-error treatment:

“I would have a lot of hope … I felt pessimistic and discouraged with each change of therapy.” [CAG-FG3-P1]

Even a negative result was often not seen as discouraging, but rather as a valuable guide to avoid ineffective options and to direct future choices.

Several emphasised that uncertainty itself was emotionally draining. Receiving a clear outcome, whether positive or negative, could reduce anxiety:

“I would be very happy because every time they changed my antidepressant, I feared that maybe it wouldn’t work.” [CAG-FG1-P1]

The ability to anticipate treatment outcomes was seen as emotionally stabilising and helpful for future decision making.

While enthusiasm for the multimodal algorithm was widespread, the discussions also revealed a range of practical concerns and emotional hesitations. As these factors have been extensively researched in the field of pharmacogenetic testing, we will keep the following sections brief.

Financial cost emerged as a prominent barrier in all focus groups, with concerns about affordability and insurance coverage. Some were willing to pay for the use of the algorithm if it shortened suffering:

“I think I would also be willing to pay something for that, because that’s just, the time, what you waste with the medications.” [MUE-FG4-P2]

Others worried about equity:

“If one doesn’t have the financial means it wouldn’t be fair.” [CAG-FG3-P4]

While the cost factor was raised by patients themselves in all focus groups, privacy was only discussed in almost all cases when prompted by the interviewer. Although most patients trusted national healthcare providers and considered data leakage to be very unlikely, they were concerned about international data sharing and potential stigmatisation:

“The way we have this standard in Germany, that it is really adhered to, so that data is not simply forwarded without anyone knowing, or that my employer suddenly knows what I have.” [MUE-FG2-P1]

Delays in receiving results were a commonly voiced concern. Long waiting periods were seen as unacceptable for individuals in acute distress, with parallels drawn to the anxiety of waiting for an HIV test:

“Then I would think, ‘in a month I’ll be crawling’.” [POZ-FG5-P2]

Many participants feared the psychological consequences of receiving a result that indicated they were not responding to antidepressants. Such a result evoked feelings of helplessness, emotional paralysis, and existential despair:

“What happens if the test really shows zero or against, what happens to the patient? You fall into an even deeper hole.” [MUE-FG1-P4]

Others feared the implications of being genetically ‘doomed’ [POZ-FG3-P3], especially in the early stages of the disease when hope is still forming:

“It just pisses me off for the reason that it kind of tips me off though, that this is something you can’t get out of.” [POZ-FG3-P3]

While family and peers were occasionally mentioned as influencing decisions, the role of information seeking was more prominent. Many patients wanted access to clear, validated scientific evidence before making a decision. Interestingly, exposure to the perspectives of other patients in the respective focus groups led some participants to change their attitudes towards the application of the algorithm. While many participants maintained their initial perspectives, others became aware of critical concerns only during the discussion. Conversely, dialogue facilitated a re-evaluation of concerns for some participants, enabling them to reconsider their reservations and become more open to testing:

“No, because at first I said I would be scared of that result, but now…. (.) I don’t know. Of course not, as we talked like that, I thought about it more.” [POZ-FG4-P1]

### 3.2 Treatment-related decision factors

Participants’ treatment histories played a major role in shaping their views on precision medicine testing. They for example pointed out that people who are just starting treatment may have a very different view of the multimodal algorithm. One recurring theme was that the algorithm should only be applied in the context of an established therapeutic relationship. Trust in the clinician was seen as essential to manage emotional responses to results:

“Maybe not at the very beginning. If you already have a good relationship with your doctor, psychiatrist or therapist who can prepare you for it.” [MUE-FG2-P1]

Few individuals who had gone through several unsuccessful treatment trials were openly pessimistic: Having *“tried wo many things, none of them helped”* [MUE-FG1-P4], they doubted that a test result could change the outcome. Some saw the multimodal algorithm as a last resort:

“I think, knowing it helps or it doesn’t help, it wouldn’t be so bad for me anymore if you say it doesn’t help, because I’m already of that opinion.” [MUE-FG2-P3]

For these individuals, even a confirmation that *“pills won’t help”* [POZ-FG5-P4] was considered useful knowledge, as it could validate their experience and redirect their efforts towards alternative treatments. Those satisfied with their current medication were less motivated to use the algorithm:

“Unlike [MUE-FG2-P3], I would now say that it works for me and that I am now quite satisfied with it and I don’t need to know whether I am imagining it or not.” [MUE-FG2-P5]

The timing within the course of illness emerged as another important consideration. Views varied on when to offer the use of the algorithm during treatment. While some advocated for testing at the start of treatment to reduce unnecessary trials, others feared that early exposure to a negative result could lead to hopelessness:

“If I heard at the beginning that the treatment I am on is not going to work, it would be worse for me.” [POZ-FG4-P1]

Mental state was mentioned as another factor influencing whether patients are willing to use the algorithm. During severe depression, some patients were more willing to accept any help available because they were desperate, while others reported being too indifferent to even consider testing at all:

“I guess it was all the same to me at the time what was going on around me.” [POZ-FG3-P3]

In contrast, they found that on better days, they were able to weigh the pros and cons more carefully and make more deliberate decisions. Ultimately, some felt that they needed external support from relatives or physicians during acute episodes because they were unable to make informed decisions on their own:

*“Sometimes even with my wife I would go to that appointment, because I kind of didn’t function, I just sat in that chair.”* [POZ-FG4-P3]

### 3.3 Underlying beliefs and perceptions influencing decisions

A key theme not widely addressed in previous research was patients’ underlying beliefs about the nature of depression and its treatment, including causal attributions, and views on recovery and medication. While some participants described depression in biological terms, others saw it as stemming from personal, social or existential experiences. These different views influenced how patients interpreted the value and relevance of the algorithm. For example, several participants expressed concern that the multimodal algorithm might oversimplify their condition by reducing it to an overly computational, standardised output that fails to take into account its psychological and individual nuances:

“Often, it seems to me that in the case of disorders such as depression, (.) it can also, however, often be an issue so individual that in the end of the day this result (.) computer may not be (.) enough.” [POZ-FG5-P4]

Some participants also noted that the use of genetic markers could overshadow the placebo effect and the importance of psychological factors:

“If you take that away completely, maybe you take away the possibility for some people, okay, maybe it would have helped, even if it’s just a lot of thinking that it helps.” [MUE-FG2-P3]

Others viewed the algorithm as an opportunity to take advantage of the placebo effect. They suggested using a placebo to support treatment through positive expectations in cases where antidepressants were likely to be ineffective.

Additionally, several participants described the social stigma associated with pharmacotherapy, expressing how “*psychotropic drugs still have this [negative] reputation in a large part of the population.*” [MUE-FG4-P4].

As a result, some participants indicated that they have avoided the use of psychotropic drugs for years for fear of dependence or physical harm. In such cases, patients pointed out that an indication of likely treatment response could provide reassurance and validate the decision to initiate pharmacological treatment:

“Maybe you have side effects, maybe it’s not always so great for the body, but I have such and such a percentage here that tells me it could help, then I think I would have tried it much sooner.” [MUE-FG2-P3]

Many patients emphasised a holistic model of care, with medication as one pillar among many. These patients, regarded antidepressants as facilitators. They enabled engagement in psychotherapy rather than being a solution in themselves. In this view, the algorithm is seen as one tool among many, the results of which should be integrated with clinical assessment and psychosocial support.

Expectations of the multimodal algorithm ranged from highly optimistic to deeply sceptical and were shaped by different understandings of what the multimodal algorithm could do, how accurate it performs, and how patients emotionally related to the idea of genetic testing. Some participants initially interpreted the algorithm as a diagnostic tool operating within a binary *“0–1 system”* [POZ-FG3-P2], while others recognised its probabilistic nature and expressed a desire for more nuanced, percentage-based results. For instance, one participant noted:

“I think living with a probability is certainly more positive than being faced with a finality.” [MUE-FG2-P1]

Another participant emphasised the importance of clarity in probabilistic feedback:

“I think that’s a margin of discretion, what probably means. Does probably mean more like 90%, so very likely or is it more like – yes, it can be but it also can’t be. So, for me a percentage result would be important.” [MUE-FG1-P1]

There was also belief among some that the multimodal algorithm could specify precise medication, and others assumed that it could diagnose depression itself, rather than merely indicate pharmacological responsiveness:

“You then also know where the sense is, yes, you now have depression. Because before endless tests are made and like that you know that immediately.” [MUE-FG4-P2]

A few patients expressed concern that the use of the algorithm might reinforce a sense of fatalism – especially if the result predicted non-response to pharmacotherapy:

“It could come out that pharmacotherapy or nothing? That there will simply be no response?” [POZ-FG3-P3]

Some also worried that too much reliance might be placed on the multimodal algorithm, ignoring individual variation. One patients stated for example:

“Maybe my biochemistry inside (.) works differently than everyone else’s. Or some little thing there that the algorithm didn’t pick up from my medical history. Or I myself did not take it into account.” [POZ-FG5-P4]

Although this argument supports the need for personalised treatment, it also highlights the concern that physicians may misinterpret the results and abandon effective treatments too soon based on a single test result. Additionally, some participants imagined that the algorithm might reveal more than what was intended, such as early signs of other medical conditions:

“They take my blood, do tests and now there is, God forbid, in my blood sample, some disease. By accident, right? And what in such a situation.” [POZ-FG5-P3]

Although often speculative, these expectations suggest that some patients perceived the multimodal algorithm as part of a broader health diagnostic tool. Many patients would only trust the algorithm if it was more than 80 or even 90 percent accurate, with some citing thresholds comparable to pregnancy tests. Lower reliability led some to prefer trial-and-error over testing:

“I don’t know, 70/30, I would find that already daring … then I’d rather try it out myself.” [MUE-FG3-P1]

This also reflects a wider concern about over-emphasising clinical data considered potentially unsafe.

The patients’ responses revealed a discrepancy between their hope that the multimodal algorithm would provide clear answers, and their fear that it might oversimplify the complexity of their disease. While many wanted precise information, others felt that overly specific, biologically framed results might alienate them or diminish the complexity of their experience:

“Too specific would probably be rather repulsive.” [MUE-FG2-P4]

Several participants raised concerns about standardising the use of the multimodal algorithm, fearing it would lead to a loss of personalisation in psychiatric care. Some argued for the integration of the algorithm into routine diagnostics, especially when patients are unable to make informed choices during acute episodes.

## 4 Discussion

This study explored patients’ perspectives and attitudes on the clinical use of a multimodal precision medicine algorithm for predicting antidepressant response, using focus group data from three European sites in the PROMPT study. While perspectives varied, most patients responded positively to the idea of a multimodal algorithm that predicts antidepressant response, which overall seems consistent with previous quantitative and qualitative research on patient attitudes to genetic and pharmacogenetic testing in psychiatry (15, 17, 24–27). Their decisions to accept or reject the use of the algorithm were shaped by a complex interplay of perceived utility, emotional readiness, previous treatment experiences and personal beliefs about depression. These results highlight the need for a nuanced, patient-centred approach to implementation into psychiatric care.

Although interest in precision medicine is increasing, it is important to recognise that the predictive power of current precision medicine tools is limited (28). In order to maintain patient trust and ensure that consent to testing is based on a realistic understanding of the clinical benefits, expectations must be carefully managed (12).

### 4.1 Recommendations for clinical communication and integration

Based on the focus group findings, we propose a set of recommendations for the patient-centred integration and communication of a multimodal algorithm for precision medicine that considers individual needs as well as considerations at the patient, physician, and healthcare system levels.

#### 4.1.1 Timing and clinical context

The timing of using the multimodal algorithm should be tailored to the stage of treatment and the emotional readiness of the patient. While patients in the early stages of the illness may benefit from early identification of an effective treatment, framing a result of non-response as a negative prognosis at an early stage may be discouraging. This reflects earlier concerns noted by Wilde et al. (29), who found that negative framing of predictive genetic test results for depression may undermine patient hope, particularly early in treatment. It also highlights the importance of clearly outlining the benefits of identifying the most suitable treatment at the outset, as well as the potential disadvantages of undergoing prolonged treatment with no guaranteed benefits. Conversely, our results align with those of Slomp et al. (11), who found that pharmacogenetic testing can offer patients with MDD who have undergone multiple unsuccessful treatments a new perspective or hope. It is important to recognise that many patients can only imagine using a multimodal algorithm in the context of a trusting therapeutic relationship, to ensure emotional support in interpreting the results.

During acute depressive episodes, consent must be approached with caution. Some patients may agree to the use of a multimodal algorithm out of desperation, but their ability to understand the implications may be limited. Consistent with the recommendations of Haga et al. (30), supported decision making, involving relatives or trusted doctors, can help to ensure that informed decisions are made.

#### 4.1.2 Communication strategies for test results

Survey studies on genetic and pharmacogenetic testing in psychiatry, including our own survey study on the PROMPT multimodal algorithm, suggest that the majority of patients are willing to undergo precision medicine testing (17, 25, 31). However, our qualitative data indicate that this enthusiasm is often based on unrealistic or false expectations. These findings highlight that survey results should be interpreted with caution and that it is important to consider acceptance rates in the context of a deeper understanding of patients’ beliefs. In line with these considerations, previous research on genetic testing in psychiatry has found that providing more detailed information about the limitations of the test can reduce interest, possibly due to more realistic expectations (29). This highlights the importance of comprehensive pre-test education, addressing misconceptions and knowledge gaps. Delivering such information effectively and ethically may require targeted training for healthcare professionals (12). Given the cognitive challenges often associated with depression, the content and delivery of information must be carefully tailored to the individual’s current mental state, ensuring that complexity and volume are adjusted accordingly.

Practitioners should emphasise that a precision medicine test such as a multimodal algorithm provides probabilistic results to inform, but not replace, clinical judgement (32). Similar to findings from previous studies on pharmacogenetic testing for MDD, our results on a multimodal algorithm revealed concerns about reductionism, which were often tied to fears of over-medication and restricted access to non-pharmacological treatment options (11, 27). Patients also varied widely regarding the level of reliability required to consider the algorithm meaningful for decision-making purposes, which highlights the importance of discussing individual expectations and thresholds for trust during the counselling process. Accordingly, the use of a multimodal algorithm should be presented as an extension of treatment options. Negative prognoses should be presented alongside clear explanations of alternatives, such as psychotherapy, neuromodulation, or lifestyle changes. Patients who wish to take antidepressants, despite their low predicted effectiveness, should have that option.

#### 4.1.3 Supporting emotional responses and patient engagement

A testing result that indicates an assumed lack of response to antidepressants may lead to despair, especially in patients who have previously been unsuccessfully treated. Clinicians need to prepare patients for this possibility and present such results constructively as an opportunity to switch to an alternative, potentially better fitting treatment, rather than as a hopeless prognosis. Emotional support before and after the use of a multimodal algorithm is crucial to minimise stress and encourage willingness to continue treatment. Offering additional interim treatments and managing expectations could also help to reduce the stress associated with delays in receiving results. In addition to the stress of long waiting times and the emotional distress of unfavourable results, several patients commented on the potential influence of expectations on treatment outcomes, reflecting awareness of placebo effects. To our knowledge, such concerns have not been previously reported from a patient perspective in the existing literature, highlighting an important area for further inquiry into how expectations shape outcomes in psychiatric care. According to Haga et al. (30), the importance patients place on genetic information, their understanding of risk and how they feel about the disease and treatment can have an effect on placebo or nocebo effects. If patients strongly believe in the credibility of a predictive multimodal algorithm, as nearly all of our participants did, they may feel hopeful or discouraged depending on the results. To manage expectations and avoid nocebo effects, communication should be tailored to patients’ needs and level of understanding (33). This might be achieved by avoiding overly deterministic language, breaking down complex genetic concepts and considering the emotional impact of the results as well as by emphasising the positive aspects related with finding the individually best fitting treatment.

These results encourage further neuroethical consideration of how biological explanations of mental illness impact patients’ sense of identity, capability, and responsibility, especially when these explanations are supported by genetic testing. The fact that some patients in our study were afraid that the outcomes of the algorithm would be more important than their personal experiences and social and emotional explanations shows how important it is to discuss not only what the multimodal algorithm can reveal, but also how patients interpret and integrate this information into their understanding of their mental health.

### 4.2 Policy and system-level considerations

In line with the overwhelming evidence from previous studies on genetic an pharmacogenetic testing in psychiatry, affordability emerged as a key barrier (8, 9, 13, 21, 25, 34, 35). Many participants indicated that they would only consider the use of a multimodal algorithm if it was covered by health insurance. Full or partial reimbursement models, possibly including co-payments or financial assistance, should be explored to promote equity. In contrast to the findings of several previous studies, concerns about data protection were rarely raised by our focus group participants (14, 17, 35). However, further probing by the interviewers revealed that this was not due to a lack of importance. Rather, it was due to the fact that high data protection standards and data compliance were taken for granted in the European countries involved. Detailed consent procedures must clarify how personal genetic data will be stored, shared and protected, especially in an international context. Policymakers must also ensure that algorithm outcomes do not restrict access to health care or justify discriminatory practices.

The results also show that the factors influencing patients’ decisions about a multimodal algorithm – such as psychological stress, beliefs, treatment history and expectations – do not act in isolation but are rather dynamically intertwined. The importance of these factors may also shift during the reflection process, as new information, supportive conversations or emotional relief may cause patients to reconsider their initial concerns or reassess the significance of the use of the algorithm. This highlights the importance of personalised counselling that considers both the need for information and the emotional and existential aspects of decision-making.

To our knowledge, this is the first study to show that the hopes and concerns that influence patients’ decisions to accept or reject a multimodal precision medicine algorithm for antidepressant efficacy are very similar to those previously reported in the context of genetic or pharmacogenetic testing. Issues such as fear of reductionism (11, 27), concerns about stigma or determinism (14, 29, 36), and emotional responses to probabilistic information (18, 22) emerged across decision contexts. This finding suggests that patients’ reactions to predictive tests are not primarily influenced by the specific type of test itself (e.g. genetic vs. algorithmic), but rather by underlying beliefs about mental illness, personal identity, and expectations about treatment, highlighting the importance of addressing these underlying dimensions consistently across different types of tests.

As pharmacogenetic tests and precision medicine tools become more widely available, including direct-to-consumer options, there is a risk that they will spread faster than the clinical guidelines and patient education (37). Our findings show the need for strong frameworks, including doctor training, clear protocols, and safeguards against misinformation.

### 4.3 Limitations

The study focused on patients with longer treatment histories of MDD and assessed hypothetical responses to a multimodal algorithm not yet in clinical use. Understanding of the algorithm may have varied between focus groups, and limitations in sample selection, including small or homogeneous groups, may affect generalisability. Furthermore, it is possible that our sample is biased by selection, as participants may have had a greater interest in research and health issues than the patient population as a whole.

Care settings varied between sites: the participants in Münster were inpatients, those in Cagliari were outpatients, and the Poznań sample included both. These contextual differences may have influenced how participants perceived the relevance, usefulness and risks of algorithmic testing. While site-specific differences were not analysed systematically, the treatment setting and illness severity are likely to have shaped the responses and should be considered when interpreting the findings.

While we ensured consistency through a consensus-based approach and iterative team validation, we did not quantify inter-coder agreement using formal metrics.

Finally, we did not collect detailed socio-demographic data relevant to technology acceptance, such as educational attainment, employment in the health or technology sectors, and prior experience with health apps or predictive testing. We are therefore unable to assess how these factors may have influenced participants’ views. Future research should consider their potential role in shaping acceptance of precision psychiatry tools.

### 4.4 Implications for future research

Future research in precision medicine in psychiatry in should include qualitative studies of healthcare professionals’ perspectives, with a particular focus on their understanding of test results and their communication approaches to patients. In addition, the experiences and attitudes of newly diagnosed patients should be considered, as they may have different information needs and emotional responses compared to patients with chronic conditions. The influence of algorithm outcomes on placebo or nocebo effects is another area that requires further research. To ensure culturally sensitive implementation, cultural differences in beliefs and expectations about mental health and genetic testing should be taken into account. Moreover, it is important to explore interventions that align patient expectations with the actual capabilities and limitations of a multimodal algorithm, to reduce misconceptions and enhance its perceived value.

Demographic factors such as age and digital literacy may also influence how patients interpret and engage with predictive tools, particularly in relation to trust and probabilistic reasoning. These potential effects should be addressed in future studies.

As our research, like most studies on pharmacogenetic testing, focuses on hypothetical decision-making processes, research into the practical implementation is particularly important. This is all the more important given that previous studies suggest that people are significantly more likely to accept genetic testing in hypothetical scenarios than in real-life situations (38). These studies should also examine how patients’ attitudes and decisions develop, and the conditions under which this occurs. They should further consider the long-term impact of treatment guided by the results of a multimodal algorithm on outcomes, treatment adherence, and patient satisfaction.

The results of our study not only contribute to improving the patient-centred implementation of predictive tests, but also raise fundamental ethical and epistemological questions about the role of using a precision medicine algorithm including genetic information in psychiatry. These includes questions such as: What knowledge about mental illness is considered most meaningful – genetic data or patients’ life experiences? How is this knowledge obtained, interpreted and communicated in clinical settings? What assumptions underlie the use of genetic testing, and is there a risk that a strictly biomedical model will be reinforced at the expense of a more holistic or psychosocial understanding? To ensure that tests empower rather than constrain patients, ongoing efforts are needed to promote education, communication and context-appropriate use.

## Data Availability

The raw data supporting the conclusions of this article will be made available by the authors, without undue reservation.
