# Supplementary material for: Factors influencing patient decision-making on a multimodal precision medicine algorithm for depression: a qualitative European multicentre study of the PROMPT consortium
Source: Front Psychiatry. 2025 Dec 16;16:1713160. doi: 10.3389/fpsyt.2025.1713160 (PMC12747837; doi:10.3389/fpsyt.2025.1713160)
Supplement: Supplementary file 1 [file Table1.docx]

**Supplemantary table 1.** Overview of the interview guide used in the PROMPT study

| **Section** | **Topic area** | **Example questions** |
| --- | --- | --- |
| **Part 1: Treatment experiences** | |  |
|  | Perceived cause of illness | What do you believe caused your depression? |
|  | Treatment history and outcomes | What forms of therapy have you received, and what worked well or less well? |
|  | Role of medication | You have all taken medication for MDD. What role did it play in your therapy? |
|  | Comparison to current treatment | How do you feel about your current therapy compared to earlier approaches? |
| **Part 2: Reactions to predictive testing** | |  |
|  | First impressions of testing | What thoughts do you have when you hear about this newly developed test? |
|  | Openness to testing | Would you feel open to it or rather hesitant? Can you elaborate? |
|  | Decision-making criteria | If you had to decide whether to take the test, what would influence your decision? |
|  | Emotional responses to test results | Let’s say you received the result – how do you think you would feel about it? |
|  | Impact on past treatment experiences | Do you think such a test would have changed anything in your previous treatment experience? |
|  | Communication and information needs | If your doctor offered you the test, what would you want to know beforehand? |
|  | Relationship with doctor | Would your willingness depend on your relationship with your doctor? |
|  | Financial aspects | Would cost play a role in your decision to take the test? |
|  | Ethical or personal beliefs | Would personal or religious beliefs affect your view of the test? |
|  | Data privacy | What do you think about data security and privacy in relation to this test? |
|  | Sharing with others | Would you tell close people about the test results?” |
|  | Motivators and barriers | What would most motivate or prevent you from agreeing to the test? |

Note. The listed questions are examples and do not represent the full interview guide.
